# Structured ICU resource management in a pandemic is associated with favorable outcome in critically ill COVID‑19 patients

**DOI:** 10.1007/s00508-020-01764-0

**Published:** 2020-11-10

**Authors:** Sebastian J. Klein, Romuald Bellmann, Hannes Dejaco, Stephan Eschertzhuber, Dietmar Fries, Wilhelm Furtwängler, Lukas Gasteiger, Walter Hasibeder, Raimund Helbok, Christoph Hochhold, Stefanie Hofer, Lukas Kirchmair, Christoph Krismer, Eugen Ladner, Georg F. Lehner, Simon Mathis, Andreas Mayr, Markus Mittermayr, Andreas Peer, Christian Preuß Hernández, Bruno Reitter, Mathias Ströhle, Michael Swoboda, Claudius Thomé, Michael Joannidis

**Affiliations:** 1 Division of Intensive Care and Emergency Medicine, Department of Internal Medicine, Medical University Innsbruck, Anichstraße 35, 6020 Innsbruck, Austria; 2 Doctoral College Medical Law and Healthcare, Faculty of Law, University Innsbruck, Innsbruck, Austria; 3 Department of Anesthesia and Critical Care Medicine, Medical University Innsbruck, Innsbruck, Austria; 4 Department of Anesthesia and Intensive Care Medicine, Hospital Hall, Hall, Austria; 5 Department of General and Surgical Intensive Care Medicine, Medical University Innsbruck, Innsbruck, Austria; 6 Department of Anesthesia and Intensive Care Medicine, Hospital Kufstein, Kufstein, Austria; 7 Department of Anesthesiology and Critical Care Medicine, Hospital St. Vinzenz Zams, Zams, Austria; 8 Department of Neurology, Medical University Innsbruck, Innsbruck, Austria; 9 Department of Internal Medicine, Hospital Hall, Hall, Austria; 10 Department of Anesthesia and Critical Care Medicine, Hospital Schwaz, Schwaz, Austria; 11 Department of Internal Medicine, Hospital St. Vinzenz Zams, Zams, Austria; 12 Department of Anesthesia and Intensive Care Medicine, Hospital Reutte, Reutte, Austria; 13 Department of Anesthesia and Intensive Care Medicine, Hospital Lienz, Lienz, Austria; 14 Department of Neurosurgery, Medical University Innsbruck, Innsbruck, Austria; 15 Department of Anesthesia and Intensive Care Medicine, Hospital St. Johann in Tyrol, St. Johann in Tyrol, Austria

**Keywords:** SARS-CoV‑2, Comorbidity, Invasive mechanical ventilation, Extracorporeal membrane oxygenation, Acute kidney injury

## Abstract

**Introduction:**

On February 25, 2020, the first 2 patients were tested positive for severe acute respiratory syndrome coronavirus‑2 (SARS-CoV-2) in Tyrol, Austria. Rapid measures were taken to ensure adequate intensive care unit (ICU) preparedness for a surge of critically ill coronavirus disease-2019 (COVID-19) patients.

**Methods:**

This cohort study included all COVID-19 patients admitted to an ICU with confirmed or strongly suspected COVID-19 in the State of Tyrol, Austria. Patients were recorded in the Tyrolean COVID-19 intensive care registry. Date of final follow-up was July 17, 2020.

**Results:**

A total of 106 critically ill patients with COVID-19 were admitted to 1 of 13 ICUs in Tyrol from March 9 to July 17, 2020. Median age was 64 years (interquartile range, IQR 54–74 years) and the majority of patients were male (76 patients, 71.7%). Median simplified acute physiology score III (SAPS III) was 56 points (IQR 49–64 points). The median duration from appearance of first symptoms to ICU admission was 8 days (IQR 5–11 days).

Invasive mechanical ventilation was required in 72 patients (67.9%) and 6 patients (5.6%) required extracorporeal membrane oxygenation treatment. Renal replacement therapy was necessary in 21 patients (19.8%). Median ICU length of stay (LOS) was 18 days (IQR 5–31 days), median hospital LOS was 27 days (IQR 13–49 days).

The ICU mortality was 21.7% (23 patients), hospital mortality was 22.6%. There was no significant difference in ICU mortality in patients receiving invasive mechanical ventilation and in those not receiving it (18.1% vs. 29.4%, *p* = 0.284). As of July 17th, 2020, two patients are still hospitalized, one in an ICU, one on a general ward.

**Conclusion:**

Critically ill COVID-19 patients in Tyrol showed high severity of disease often requiring complex treatment with increased lengths of ICU and hospital stay. Nevertheless, the mortality was found to be remarkably low, which may be attributed to our adaptive surge response providing sufficient ICU resources.

**Electronic supplementary material:**

The online version of this article (10.1007/s00508-020-01764-0) contains supplementary material, which is available to authorized users.

## Introduction

Since the first cases of coronavirus disease 2019 (COVID-19) in Wuhan, China in December 2019, the severe acute respiratory syndrome coronavirus 2 (SARS-CoV-2) has caused a pandemic [1]. In Lombardy, Italy, one of the most severely affected regions in Europe, the first patient tested positive for SARS-CoV‑2 on 20 February 2020 [2]. The federal province of Tyrol, Austria, borders northern Italy and hosts the most used transit route between Italy and Germany. Tyrol has approximately 750,000 inhabitants and during winter season is additionally heavily populated by over 300,000 winter tourists [3]. On 25 February 2020, the first 2 patients were tested positive in Innsbruck, Tyrol, Austria.

In response to alarming experiences in northern Italy, a rapid coordination between intensive care specialists was quickly established, facilitating allocation plans for critically ill COVID-19 patients in Tyrol. Approximately 183 intensive care unit (ICU) beds are available in Tyrol under regular conditions [4]. Eight ICUs in secondary hospitals mainly served as primary treatment centers, while patients requiring tertiary care or extracorporeal membrane oxygenation (ECMO) were transferred by specialized infectious diseases intensive care transport providers to the University Hospital in Innsbruck, Tyrol, Austria for further treatment.

The aim of this study was to evaluate baseline characteristics, treatment and outcomes of critically ill COVID-19 patients in Tyrol, managed by a structured approach to ICU allocation. Patients were registered in the Tyrolean COVID-19 intensive care registry (Tyrol-CoV-ICU-Reg).

## Methods

### Patients

All patients with polymerase chain reaction (PCR) confirmed COVID-19 or strongly suspected COVID-19 infection by radiologic pulmonary findings admitted to an ICU in all hospitals in Tyrol, Austria were included in this analysis. Eight ICUs from local hospitals and five dedicated COVID-19 ICUs from the University Hospital Innsbruck, Tyrol, Austria provided data for this registry (a list of all ICUs is available in the electronic supplemental material, ESM). Patients were eligible for inclusion into the Tyrol-CoV-ICU-Reg if they were admitted to a study ICU or intermediate care unit (IMCU) between February 1 and July 17, 2020, and had confirmed COVID-19, either by SARS-CoV‑2 PCR and/or strong clinical suspicion and COVID-19 typical findings in chest computed tomography (CT). No age restrictions were applied.

Acute kidney injury (AKI) was defined and staged according to Kidney Disease: Improving Global Outcome (KDIGO) using both creatinine and urine output criteria. Comprehensive laboratory values were available for patients treated at the University Hospital Innsbruck and were provided as highest and/or lowest value where appropriate.

Date of final follow-up was July 17, 2020. The registry was approved by the local ethics committee (Nr. 1099/2020).

### Patient allocation

Patients were either primarily admitted to a regional hospital or to the University Hospital Innsbruck if they were within its catchment area. All primary hospitals provided COVID-19 ICU treatment for at least 24 h as long as sufficient capacity was available. If regional reserve capacity was below 10% or patients were in such a severe condition that a transfer in a tertiary center seemed beneficial (e.g. multiple organ failure, ECMO requirement), a transfer was organized. Transfer from peripheral hospitals to the center was coordinated by a dedicated COVID-19-ICU coordinator. The ICU capacity in the University Hospital Innsbruck was organized by the principle of avoiding an ICU occupancy rate of more than 80%. Maximum capacity of COVID-19 dedicated ICUs ranged from 10–16 beds. As soon as the occupancy rate of the most recently dedicated COVID-19 ICU was surpassing 50%, another ICU was prepared to be opened as the next COVID-19 isolation unit. Elective surgery was reduced on March 15, 2020 by an overall of 65%, but at least 50% of surgical/trauma ICU beds were kept available for treatment of non-COVID-19 patients. Hence, urgent procedures including the bulk of the transplantation program, cardiac surgical operations, trauma surgery and general surgical procedures were unaffected. Based on reports in the neighboring region of Lombardy, Italy, where on March 15, 2020 767 patients were treated concomitantly on ICU [5], COVID-19 ICU capacity in Tyrol was continuously adapted to the surge of patients with a maximum target of 129 beds available for a population of 750,000 people [6].

### Statistical analysis

Categorical variables are presented as numbers (percentage). Continuous variables are presented as median and interquartile range (IQR). Odds ratios (OR) are presented with 95% confidence interval (95% CI). Normal distribution of continuous data was checked by the Shapiro-Wilk test. Normally distributed data was compared using a Welch’s two sample t‑test. Not normally distributed data was compared using a Mann-Whitney‑U test or χ^2^-test.

Patients who were older than median age were classified as older, whereas patients below median age were classified as younger.

Statistical significance was defined as *p* < 0.05 and all statistical tests were 2‑sided. Statistical analysis was performed using R (R Foundation for Statistical Computing, Vienna, Austria).

## Results

### Patient characteristics

**Fig. 1:**
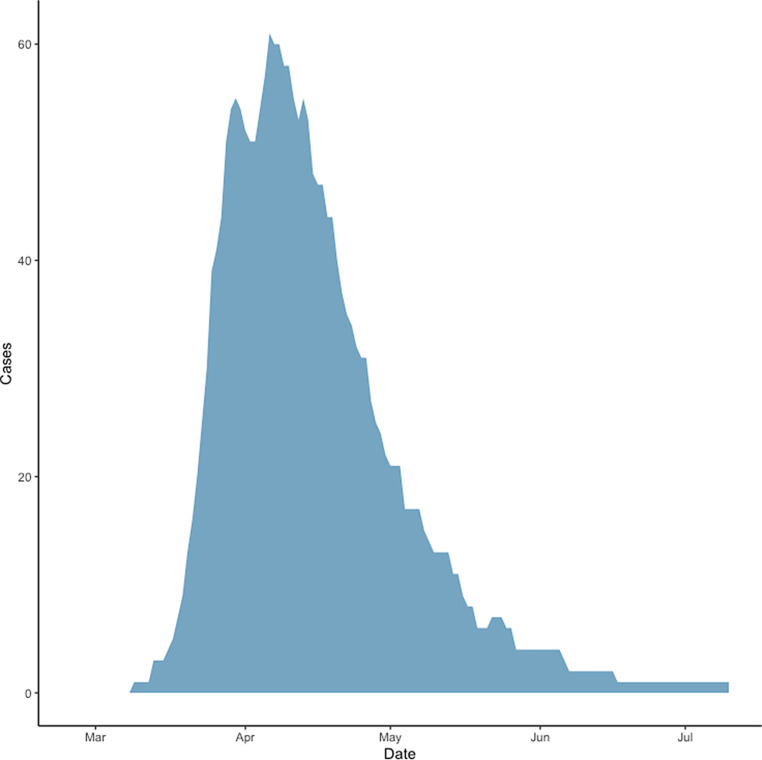
Time course of active ICU patients in Tyrol, Austria

**Fig. 2:**
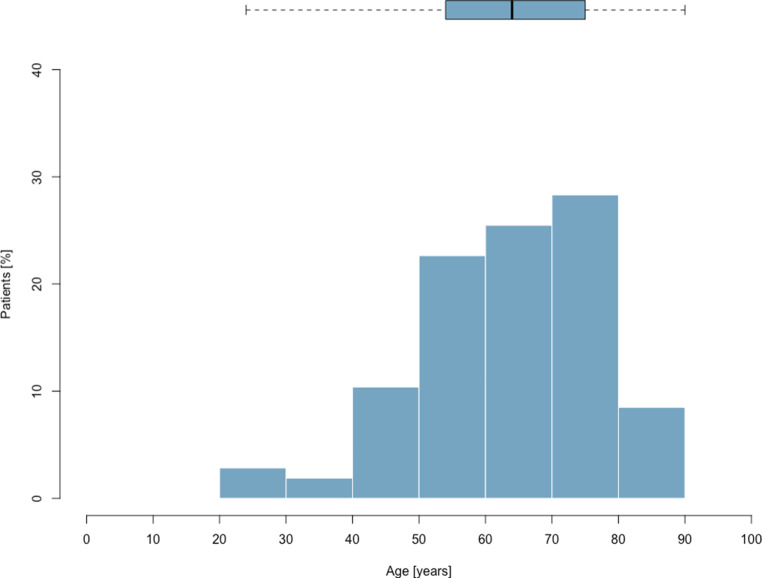
Age distribution of full cohort (*n* = 106)

From March 9 to July 17, 2020, 106 patients with COVID-19 were admitted to an ICU (Fig. 1). In total, 3596 patients tested positive for SARS-CoV‑2 in Tyrol until July 17, 2020 [7]. On April 6, 2020 a maximum of 61 patients (3.6% of active cases in Tyrol on this day) were treated in ICUs in Tyrol concurrently. The last patient was admitted on June 4, 2020. Patients’ age ranged from 24 years to 90 years, with a median age of 64 years (Fig. 2). When classifying patients according to median age 41 (38.7%) were older patients (i.e. more than 64 years old). The majority of patients were male (76 patients, 71.7%) and all patients were European. Further patient characteristics are shown in Table 1.

**Table 1:** Patient characteristics

	Overall	ICU survivors	ICU non-survivors	*p*
*n*	106	83	23	–
*Age (years, median [IQR])*	64.00 [54.00, 74.50]	63.00 [53.00, 71.00]	76.00 [69.00, 82.50]	<0.001
*Male (%)*	76 (71.7)	62 (74.7)	14 (60.9)	0.298
*BMI [kg/m*^*2*^*] (median [IQR])*	26.83 [25.07, 29.95]	26.30 [24.49, 29.39]	28.65 [26.42, 30.90]	0.041
*HbA1c [%] (median [IQR])*	6.20 [5.70, 6.70]	6.10 [5.70, 6.65]	6.50 [6.05, 6.73]	0.168
*Pregnant (%)*	0 (0.0)	0 (0.0)	0 (0.0)	NA
*European (%)*	106 (100.0)	83 (100.0)	23 (100.0)	1.000
*Patient from nursing home (%)*	0 (0.0)	0 (0.0)	0 (0.0)	NA
*Legal guardian (%)*	2 (1.9)	1 (1.2)	1 (4.3)	0.909
*Admission state (%)*	–	–	–	0.002
Fully independent daily living	91 (85.8)	75 (90.4)	16 (69.6)	–
Requires some assistance in daily activities	12 (11.3)	8 (9.6)	4 (17.4)	–
Requires full assistance in daily activities	3 (2.8)	0 (0.0)	3 (13.0)	–
*Admission from (%)*	–	–	–	0.870
Emergency room	29 (27.4)	23 (27.7)	6 (26.1)	–
General ward (diff. hospital)	1 (0.9)	1 (1.2)	0 (0.0)	–
General ward (same hospital)	65 (61.3)	49 (59.0)	16 (69.6)	–
ICU (diff. hospital)	8 (7.5)	7 (8.4)	1 (4.3)	–
ICU (same hospital)	2 (1.9)	2 (2.4)	0 (0.0)	–
Other facility	1 (0.9)	1 (1.2)	0 (0.0)	–
*SARS-CoV‑2 PCR positive (%)*	104 (98.1)	81 (97.6)	23 (100.0)	1.000
*COVID-19 typical findings in chest X‑ray (%)*	97 (92.4)	76 (92.7)	21 (91.3)	1.000
*COVID-19 typical findings in chest X‑ray (%)*	51 (81.0)	42 (85.7)	9 (64.3)	0.157
*COVID-19 primary reason for hospital admission (%)*	90 (84.9)	70 (84.3)	20 (87.0)	1.000
*SAPS III (median [IQR])*	56.00 [49.00, 64.00]	54.00 [47.00, 62.00]	63.00 [55.50, 73.00]	0.001
*First symptom to hospital admission—days (median [IQR])*	6.00 [4.00, 8.00]	7.00 [4.00, 9.00]	4.00 [2.25, 6.00]	0.002
*First symptom to ICU admission—days (median [IQR])*	8.00 [5.00, 11.00]	8.00 [6.00, 11.00]	6.00 [4.00, 8.00]	0.009
*Hospital LOS (median [IQR])*	27.00 [14.25, 41.75]	30.00 [21.00, 49.00]	9.00 [6.00, 22.50]	<0.001
*ICU LOS (median [IQR])*	18.50 [5.25, 31.75]	21.00 [11.00, 33.00]	6.00 [4.00, 20.00]	0.012
*Death in ICU (%)*	23 (21.7)	0 (0.0)	23 (100.0)	<0.001
*Death in hospital (%)*	24 (22.6)	1 (1.2)	23 (100.0)	<0.001

*ICU* intensive care unit, *IQR* interquartile range, *BMI* body mass index, *SAPS* simplified acute physiology score, *COVID-19* coronavirus disease 2019, *SARS-CoV‑2* severe acute respiratory syndrome coronavirus 2, *PCR* polymerase chain reaction, *LOS* length of stay, *HbA1c* glycated hemoglobin, *diff.* different

The majority of patients were primarily admitted to a general ward before being transferred to an ICU, while one third was admitted from an emergency room (Table 1). Of the patients, 9 (8.5%) were transferred from South Tyrol, Italy.

A total of 20 patient transfers between ICUs took place, 17 patients (16.0%) were transferred from a peripheral hospital to the University Hospital of Innsbruck, 1 patient (0.9%) was transferred from 1 peripheral hospital to another and 1 patient (0.9%) was transferred back from the University Hospital of Innsbruck to a peripheral hospital. One patient (0.9%) was readmitted to an ICU after having been primarily discharged to a general ward.

The SARS-CoV‑2 PCR was positive in 104 patients (98.1%); however, 2 patients had no positive PCR but there was strong clinical suspicion and radiological findings typical for COVID-19 in chest computed tomography (CT) and no other etiology was likely. COVID-19 was the leading cause for hospitalization in 90 patients (84.9%).

Median duration from appearance of first symptoms to hospital admission was 6 days (IQR 4–8 days) and 8 days (IQR 5–11 days) to ICU admission (Table 1).

### Comorbidities/risk factors

The most frequently observed comorbidity was arterial hypertension (71 patients, 67.0%), followed by cardiovascular (45 patients, 42.5%) and renal (21 patients, 19.8%) comorbidities. While arterial hypertension seemed to have minor impact on mortality (OR 1.64 [95% CI 0.61–4.94]) in univariate analysis, both cardiovascular (OR 4.68 [95% CI 1.79–13.37]) and renal (OR 6.09 [95% CI 2.17–17.69]) comorbidities appeared to be major risk factors for ICU mortality. Respiratory comorbidities were observed in 34 patients (32.1%), with 14 patients (13.2%) having chronic obstructive pulmonary disease (COPD) and 7 patients (6.6%) having asthma (ESM Table 1). Obesity (body mass index, BMI >30 kg/m^2^) was noticed in 24 patients (22.6%), of these, 1 patient (0.9%) was considered morbidly obese (BMI >40 kg/m^2^). History of diabetes mellitus (DM) type II was known in 16 patients (15.1%), while prediabetes and DM type I were relatively uncommon. Of the patients, 17 (16.0%) had no known comorbidities before ICU admission. Patients without comorbidities were significantly younger (53 years [36–56 years] vs. 68 years [59–76 years]; *p* < 0.001). Despite the fact that requirement of IMV was as frequent as in the total cohort (70.6% vs. 67.4%; *p* = 1.000) and 2 of the 17 patients required ECMO, they all survived.

Before being admitted to an ICU because of COVID-19, most patients (91, 85.8%) were considered to be fully independent in daily activities, while 12 patients (11.3%) required some assistance and 3 patients (2.8%) required full assistance in daily activities. Patients who were not fully independent in daily activities showed significantly higher hospital mortality (*p* = 0.001).

Comprehensive laboratory reports were available for 49 patients, who were treated at the University Hospital Innsbruck (ESM Table 2).

### Treatment

Overall, 72 patients (67.9%) received IMV. Before commencing IMV, one or several noninvasive respiratory support measures were initially provided, namely noninvasive ventilation (NIV) in 52 patients (72.3%), nasal high flow (NHF) in 15 patients (20.8%) and oxygen mask in 55 (76.4%) patients.

Of the 34 patients (32.1%) who ultimately did not receive IMV, 27 patients (79.4%) received NIV, 8 patients (23.5%) received NHF and 32 (94.1%) received an oxygen mask.

Prone positioning was performed in 58 patients (54.7%). Muscle relaxation to facilitate respiratory support was required in 26 patients (24.5%) intermittently, while 10 patients (9.4%) required continuous muscle relaxation. Vasopressors were required in 74 patients (69.8%). They were significantly more often required in mechanically ventilated patients (Table 2).

**Table 2:** Patient characteristics grouped by receipt of invasive mechanical ventilation (IMV)

	Overall	No IMV	IMV	*p*
*n*	106	34	72	–
*Age (years median [IQR])*	64.00 [54.00, 74.50]	68.00 [54.75, 77.25]	63.50 [54.00, 72.00]	0.280
*Male (%)*	76 (71.7)	23 (67.6)	53 (73.6)	0.685
*BMI (median [IQR])*	26.83 [25.07, 29.95]	25.97 [24.31, 28.04]	27.18 [25.18, 30.85]	0.118
*SAPS III (median [IQR])*	56.00 [49.00, 64.00]	53.50 [46.00, 62.00]	57.00 [49.50, 65.50]	0.062
*Admission state (%)*	–	–	–	0.025
Fully independent daily living	91 (85.8)	26 (76.5)	65 (90.3)	–
Required some assistance in daily activities	12 (11.3)	5 (14.7)	7 (9.7)	–
Required full assistance in daily activities	3 (2.8)	3 (8.8)	0 (0.0)	–
*Death in ICU (%)*	23 (21.7)	10 (29.4)	13 (18.1)	0.284
*Death in hospital (%)*	24 (22.6)	10 (29.4)	14 (19.4)	0.370
*ICU LOS [days] (median [IQR])*	18.50 [5.25, 31.75]	5.00 [3.00, 6.75]	25.00 [18.00, 35.25]	<0.001
*Hospital LOS [days] (median [IQR])*	27.00 [13.25, 49.50]	10.50 [7.00, 18.75]	35.50 [24.75, 64.50]	<0.001
*AKI (%)*	–	–	–	<0.001
No AKI	54 (50.9)	29 (85.3)	25 (34.7)	–
KDIGO I	16 (15.1)	2 (5.9)	14 (19.4)	–
KDIGO II	9 (8.5)	0 (0.0)	9 (12.5)	–
KDIGO III	27 (25.5)	3 (8.8)	24 (33.3)	–
*RRT (%)*	21 (19.8)	1 (2.9)	20 (27.8)	0.006
*Vasopressors (%)*	74 (69.8)	7 (20.6)	67 (93.1)	<0.001
*Prone positioning (%)*	58 (54.7)	1 (2.9)	57 (79.2)	<0.001

*IMV* invasive mechanical ventilation, *IQR* interquartile range, *BMI* body mass index, *SAPS* simplified acute physiology score, *ICU* intensive care unit, *LOS* length of stay, *AKI* acute kidney injury, *KDIGO* kidney disease: improving global outcomes, *RRT* renal replacement therapy

**Table 3:** Interventions, length of interventions and frequency of do not resuscitate (DNR) or best supportive care orders

	Overall	ICU survivors	ICU nonsurvivors	*p*
*n*	106	83	23	–
*IMV (%)*	72 (67.9)	59 (71.1)	13 (56.5)	0.284
NIV before IMV (%)	52 (72.3)	44 (74.6)	8 (34.8)	0.534
NHF before IMV (%)	15 (20.8)	14 (23.7)	1 (4.3)	0.364
Oxygen before IMV (%)	55 (76.4)	45 (76.3)	10 (43.5)	1.000
*No IMV (%)*	34 (32.1)	24 (28.9)	10 (43.5)	0.284
NIV (no IMV) (%)	27 (79.4)	18 (75.0)	9 (90.0)	0.603
NHF (no IMV) (%)	8 (23.5)	6 (25.0)	2 (20.0)	1.000
Oxygen (no IMV) (%)	32 (94.1)	22 (91.7)	10 (100.0)	0.888
*Prone positioning (%)*	58 (54.7)	47 (56.6)	11 (47.8)	0.608
*Muscle relaxation (%)*	–	–	–	0.336
No muscle relaxation	70 (66.0)	56 (67.5)	14 (60.9)	–
Intermittent muscle relaxation	26 (24.5)	21 (25.3)	5 (21.7)	–
Continuous muscle relaxation	10 (9.4)	6 (7.2)	4 (17.4)	–
*Vasopressors (%)*	74 (69.8)	55 (66.3)	19 (82.6)	0.210
*AKI (%)*	–	–	–	0.097
No AKI	54 (50.9)	44 (53.0)	10 (43.5)	–
KDIGO I	16 (15.1)	15 (18.1)	1 (4.3)	–
KDIGO II	9 (8.5)	7 (8.4)	2 (8.7)	–
KDIGO III	27 (25.5)	17 (20.5)	10 (43.5)	–
*RRT (%)*	21 (19.8)	17 (20.5)	4 (17.4)	0.973
*vv-ECMO (%)*	6 (5.7)	5 (6.0)	1 (4.3)	1.000
*IMV [days] (median [IQR])*	15.00 [10.00, 23.25]	15.00 [10.50, 22.00]	15.00 [7.00, 31.00]	0.959
*NIV [days] (median [IQR])*	3.00 [1.00, 6.00]	4.00 [1.00, 6.25]	2.00 [1.00, 4.00]	0.068
*NHF [days] (median [IQR])*	1.00 [1.00, 1.50]	1.00 [1.00, 2.00]	1.00 [1.00, 1.00]	0.314
*Prone positioning [days] (median [IQR])*	4.00 [2.00, 5.75]	4.00 [2.00, 5.50]	4.00 [2.50, 5.50]	0.695
*Continuous muscle relaxation [days] (median [IQR])*	1.00 [1.00, 2.00]	1.00 [1.00, 1.00]	2.00 [1.00, 3.00]	0.039
*RRT [days] (median [IQR])*	11.00 [3.00, 24.00]	10.00 [2.00, 20.00]	21.50 [9.50, 34.00]	0.318
*vv-ECMO [days] (median [IQR])*	12.00 [11.25, 14.25]	12.00 [11.00, 15.00]	12.00 [12.00, 12.00]	1.000
*DNR order*	26 (24.5)	7 (8.4)	19 (82.6)	<0.001
DNR—no CPR (%)	22 (20.7)	4 (4.8)	18 (78.3)	<0.001
DNR—no (re-)intubation (%)	10 (9.4)	2 (2.4)	8 (34.8)	<0.001
DNR—no ECMO (%)	17 (16.0)	6 (7.2)	11 (47.8)	<0.001
DNR—other (%)	13 (12.3)	2 (2.4)	11 (47.8)	<0.001
*Best supportive care (%)*	13 (12.3)	1 (1.2)	12 (52.2)	<0.001

*ICU* intensive care unit, *IMV* invasive mechanical ventilation, *NIV* noninvasive ventilation, *NHF* nasal high flow, *AKI* acute kidney injury, *KDIGO* Kidney disease: improving global outcomes, *RRT* renal replacement therapy, *vv-ECMO* veno-venous extracorporeal membrane oxygenation, *IQR* interquartile range, *DNR* do not resuscitate, *CPR* cardiopulmonary resuscitation

**Fig. 3:**
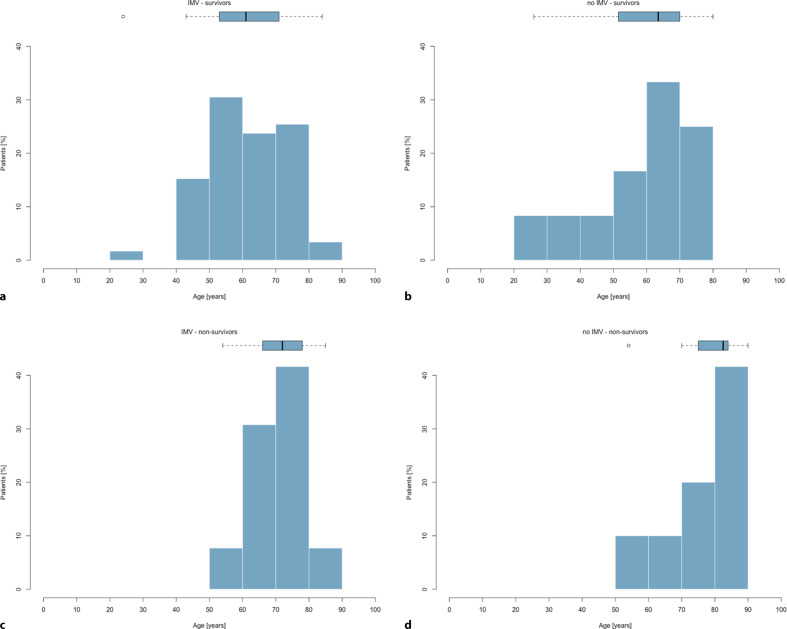
**a** Age of patients receiving IMV who survived ICU stay, **b** age of patients who did not receive IMV who survived ICU stay, **c** age of patients receiving IMV who did not survive ICU stay, **d** age of patients who did not receive IMV and who did not survive ICU stay

Patients receiving IMV had no significant difference in SAPS III scores compared to patients not receiving IMV but had more AKI and required RRT significantly more often. There was no significant difference in ICU mortality between patients who received IMV and those who did not (Table 2; Fig. 3).

Median duration of IMV was 15 days (IQR, 10–24 days). Duration was considerably shorter for NIV (3 days [IQR, 1–6 days]) and NHF (1 day [IQR, 1–2 days]). While there was no difference in do not resuscitate (DNR)—no cardiopulmonary resuscitation (CPR) orders between patients receiving IMV and no IMV (13/72 [18.1%] vs. 9/34 [26.5%]; *p* = 0.459), there was a significant difference in DNE—no (re-)intubation orders (1/72 [1.4%] vs. 9/34 [26.5%]; *p* < 0.001).

Veno-venous ECMO (vv-ECMO) was performed in six patients (5.7%). One patient requiring ECMO died, all other patients were successfully weaned from vv-ECMO. Medium SAPS III of ECMO patients was 59 (IQR, 48–79) as compared to 56 (IQR, 49–64) in patients not requiring ECMO (*p* = 0.576). Four patients receiving vv-ECMO developed AKI, while one of those patients required RRT. Medium duration of vv-ECMO was 12 days (IQR 11–14 days).

Almost half (49.1%) of the patients in our cohort developed AKI according to the KDIGO criteria. The majority had KDIGO stage III (27 patients, 25.5%), while 9 patients (8.5%) had KDIGO stage II and 16 patients (15.1%) had KDIGO stage I AKI. While RRT was administered in 21 patients (19.8%), it was required in 20 patients (18.9%) due to AKI and in 1 patient due to end stage renal disease (ESRD). Median duration of RRT was 11 days (IQR, 3–24 days).

Favipiravir was administered in 39 patients (36.8%). Admission SAPS III did not differ significantly between patients who received favipiravir and who did not (56.5 [IQR, 49.25–63.75] vs. 55.0 [IQR, 46.5–64.5]; *p* = 0.570). There was no significant difference in ICU mortality (7/34 [20.6%] vs. 4/27 [10.8%]; *p* = 0.418) or ICU (33.5 days [IQR, 23.0–45.4 days] vs. 37.0 days [IQR, 28.0–56.0 days]; *p* = 0.157) or hospital length of stay (LOS) (24.5 days [IQR, 17.3–38.8 days] vs. 25.0 days [IQR, 21.0–34.0 days]; *p* = 0.945).

Hydroxychloroquine was administered in 54 patients (50.9%). There was no significant difference in median SAPS III scores (56.5 [IQR, 49.3–63.8] vs. 55.0 [IQR, 46.5–64.5]; *p* = 0.570). Patients with hydroxychloroquine received IMV significantly more often (45/54 [83.4%] vs. 26/52 [50%]; *p* = 0.001). They had significantly more often AKI (35/54 [64.8%] vs. 17/52 [32.7%]; *p* = 0.009).

### Outcome

A total of 23 patients (21.7%) died in the ICU, while 1 patient (0.9%) died after ICU discharge on a general ward.

Median hospital length of stay was 27 days (IQR, 14–42 days), while ICU length of stay was 18 days (IQR, 5–32 days). Survivors had significantly longer hospital and ICU length of stay (Table 1).

Patients who died were significantly older than ICU survivors (ESM Table 3, ESM Fig. 1). They also had a significantly shorter duration between first symptoms and hospital and ICU admission. There was a tendency to higher BMI in patients who died in ICU (Table 3).

As of 17 July 2020, one patient (0.9%) is still in ICU, a second patient requires treatment on a normal ward. All other remaining patients were discharged from hospital.

## Discussion

This is a comprehensive report of all 106 critically ill COVID-19 patients treated in ICUs in Tyrol following a locally established adaptive surge response model. Tyrol is a small federal province in Austria, which was hit quite hard and early by COVID-19 due to its spread through winter tourism. The peak in ICU occupancy rate was reached on 6 April, with a maximum of 61 patients. Whereas age distribution and severity of illness was quite comparable to reports for other regions [2, 8], outcome was remarkably good with an ICU mortality of 21.7% and a total hospital mortality of 22.6%.

The majority of patients were male, median age was 64 years. Frequently observed comorbidities in our cohort were arterial hypertension, cardiovascular disease and chronic kidney disease. Pulmonary comorbidities were only present in few patients. This is in line with previous reports [2, 9]. History of diabetes mellitus was mainly restricted to type II diabetes and relatively infrequent as compared to other reports [10–12]; however, as we recently demonstrated, many patients suffered from unrecognized diabetes mellitus on ICU admission [13].

IMV was necessary in 67.9% of patients which is in accord with previously reported rates ranging from 48% to 76% [2, 9, 14, 15]. Duration of IMV was relatively long with a median of 15 days. Likewise, median ICU and hospital length of stay were 18 days and 27 days, respectively. Following our internal guideline for ICUs in Tyrol, which recommended a stepwise approach for respiratory support in COVID-19 patients, most patients received a trial of noninvasive ventilation techniques before IMV, starting with NHF limited to a maximum flow of 20 L/min followed by NIV if NHF was insufficient to achieve adequate oxygenation. Hospital mortality of IMV patients was 19.4%, which is much lower than a recently published report form Germany [9]. Furthermore, there was no significant difference in mortality between patients receiving IMV and those who did not. Possible explanations may be lower median age of Tyrolean IMV patients compared to Germany as well as patient selection due to the fact that rate of DNE orders was higher in patients who received NIV only [9]. We strictly applied the recommended approach of lung protective ventilation and initiated ECMO in case this was not possible according to ELSO criteria [16, 17]. With 5.6% of patients receiving vv-ECMO, rate of use of vv-ECMO was considerably higher than other reports [2]. Interestingly, nearly all patients recovered their lung function and could be weaned from ECMO, indicating that early use of this technique may result in improved outcome in COVID-19 associated ARDS. Similar findings have previously been reported for other forms of viral pneumonias, e.g. during the H1N1 pandemic [18].

Most patients received at least one antiviral substance. The most commonly used substances were favipiravin and hydroxychloroquine. Remdesivir was not available in Tyrol during the surge. We did not observe an effect of favipiravin on mortality or ICU or hospital length of stay; however, patients receiving hydroxychloroquine had higher rates of AKI. There was no significant difference in ICU mortality for hydroxychloroquine. Besides selected antiviral therapies, we did not deploy experimental methods, e.g. plasmapheresis or antibodies interfering with IL‑6 pathway. Steroids were not given routinely before the publication of the RECOVERY trial [19].

Previously reported rates of AKI in critically ill COVID-19 patients were mostly relatively low, with 2 meta-analysis showing a pooled incidence of 11% and 36.4%,r respectively [20, 21]; however, when strictly applying the KDIGO creatinine and urine output criteria, we observed a high rate of AKI and RRT in our cohort. Possible explanations for AKI in COVID-19 include direct effects of lung injury or IMV on the kidneys [22], but AKI also may be due to direct effects caused by SARS-CoV‑2 [20].

Despite a substantial severity of disease of our ICU patients, which is quite similar to published cohorts, ICU mortality was remarkably low in our cohort with 21.7%. In line with previous observations, mortality was higher in older patients and in patients with higher frailty [23]. Of note, nearly all patients who were discharged alive from ICU were also discharged alive from hospital. Even when assuming that the remaining two patients still hospitalized would not survive, the resulting ICU and hospital mortalities of 22.6% and 23.5%, respectively, would still be lower than most previous reports for critically ill COVID-19 patients. Furthermore, reported mortality rates ranging from 26% to 88% [2, 8, 24–27] include many patients still being treated in hospitals.

We can only speculate about the reasons for this favorable outcome. First of all, the cohort of mechanically ventilated patients comprising two thirds of all our patients was relatively young. A recently published large cohort from Germany reported a median age of 70.0 years versus 63.5 years in our cohort [9]. Other factors may be a local case mix different to other cohorts, high socioeconomic status in our region and a highly developed and easily accessible healthcare system in Austria. Very likely, though, our approach of a structured ICU resource management during the COVID-19 surge may have significantly contributed to low mortality.

By acknowledging early experiences from Lombardy, Italy, rapid measures were taken to prepare ICUs in Tyrol, Austria for a surge of critically ill COVID-19 patients. A coordinating network of intensive care specialists was established to avoid uncoordinated patient movements between ICUs and to evenly distribute patients according to medical demands (e.g. requirement of ECMO) or in terms of resource management (e.g. available ICU beds). Any requests for transfer were managed by a central coordinator at the University Hospital Innsbruck. Our approach can be regarded as a principally decentralized patient allocation but with a central institution providing coordination and back-up. Whether such a decentralized approach to patient distribution is truly beneficial remains to be proven; however, we experienced a relatively equal degree of ICU occupancy across all centers, providing enough free ICU beds for critically ill COVID-19 and non-COVID-19 patients at all times. With a maximum of 61 concurrent COVID-19 ICU patients, only 47.3% of our planned capacity of 129 COVID-19 ICU beds was utilized. Therefore, we were never forced to employ triage measurements, limiting ICU access only to younger patients with fewer comorbidities, as it was reported for some regions [28, 29].

Our report has several strengths. Firstly, all COVID-19 patients who required ICU admission in a defined region were included, full registration of all patient characteristics was cross-checked by patient transfer data. Secondly, no patient was lost to follow-up enabling reliable outcome data and finally, ICU bed availability and occupancy rates of ICUs treating COVID-19 patients were recorded over the whole period.

Some limitations must be noted. While we included all COVID-19 ICU patients, data from COVID-19 patients on general wards, who ultimately did not require or receive ICU treatment were not collected. Therefore, we can only speculate on pre-ICU admission patient selection. Also, compared to other published cohorts, our study population was of relatively small size; however, we were capable of including all ICU patients of all hospitals in a closed region of 750,000 inhabitants, therefore providing a comprehensive cross-section of critically ill COVID-19 patients.

## Conclusion

This report provides a comprehensive summary of all critically ill COVID-19 patients treated in Tyrol, Austria, under provision of an adaptive surge response. Whereas patients were severely ill with high SAPS III scores, high rates of respiratory failure as well as AKI, long durations of ICU and hospital stay, mortality was remarkably low. Avoidance of ICU overload in combination with early lockdown measures may have played a significant role in this favorable outcome.

## Caption Electronic Supplementary Material

A list of COVID-19 ICUs who participated in the Tyrol-CoV-ICU-Reg and ESM Tables 1–3 and ESM Figure 1 are provided in the electronic supplemental material

## References

[1] Wang C, Horby PW, Hayden FG, Gao GF (2020). A novel coronavirus outbreak of global health concern. Lancet.

[2] Grasselli G, Zangrillo A, Zanella A, Antonelli M, Cabrini L, Castelli A, et al. Baseline Characteristics and Outcomes of 1591 Patients Infected With SARS-CoV‑2 Admitted to ICUs of the Lombardy Region, Italy. JAMA. 2020;323(16):1574–81. 10.1001/jama.2020.5394. 10.1001/jama.2020.5394 PMC7136855 32250385

[3] Tourism in Tyrol. https://www.tirol.gv.at/statistik-budget/statistik/tourismus/. Accessed: 02 July 2020.

[4] Documentation of the LKF hospitals Austria. 2018. http://www.kaz.bmg.gv.at/fileadmin/user_upload/Publikationen/Oesterreich_2018.pdf. Accessed: 02 July 2020.

[5] Bulletin Italian Ministry of Health. 2020. http://www.salute.gov.it/imgs/C_17_pagineAree_5351_14_file.pdf. Accessed: 02 July 2020.

[6] Aziz S, Arabi YM, Alhazzani W, Evans L, Citerio G, Fischkoff K (2020). Managing ICU surge during the COVID-19 crisis: rapid guidelines. Intensive Care Med.

[7] Coronavirus COVID-19 Dashboard Tyrol. https://www.tirol.gv.at/dashboard. Accessed: 10 July 2020.

[8] Bhatraju PK, Ghassemieh BJ, Nichols M, Kim R, Jerome KR, Nalla AK (2020). Covid-19 in critically ill patients in the Seattle region—case series. N Engl J Med.

[9] Karagiannidis C, Mostert C, Hentschker C, Voshaar T, Malzahn J, Schillinger G (2020). Lancet Respir Med.

[10] Apicella M, Campopiano MC, Mantuano M, Mazoni L, Coppelli A, Del Prato S (2020). COVID-19 in people with diabetes: understanding the reasons for worse outcomes. Lancet Diabetes Endocrinol.

[11] Cariou B, Hadjadj S, Wargny M, Pichelin M, Al-Salameh A, Allix I (2020). Phenotypic characteristics and prognosis of inpatients with COVID-19 and diabetes: the CORONADO study. Diabetologia.

[12] Barron E, Bakhai C, Kar P, Weaver A, Bradley D, Ismail H (2020). Associations of type 1 and type 2 diabetes with COVID-19-related mortality in England: a whole-population study. Lancet Diabetes Endocrinol.

[13] Klein SJ, Fries D, Kaser S, Mathis S, Thome C, Joannidis M (2020). Unrecognized diabetes in critically ill COVID-19 patients. Crit Care.

[14] Dreher M, Kersten A, Bickenbach J, Balfanz P, Hartmann B, Cornelissen C (2020). The characteristics of 50 hospitalized COVID-19 patients with and without ARDS. Dtsch Arztebl Int.

[15] Auld SC, Caridi-Scheible M, Blum JM, Robichaux C, Kraft C, Jacob JT (2020). ICU and ventilator mortality among critically ill adults with Coronavirus disease 2019. Crit Care Med..

[16] Bartlett RH, Ogino MT, Brodie D, McMullan DM, Lorusso R, MacLaren G (2020). Initial ELSO guidance document: ECMO for COVID-19 patients with severe cardiopulmonary failure. ASAIO J.

[17] Wiedemann D, Bernardi MH, Distelmaier K, Goliasch G, Hengstenberg C, Hermann A (2020). Wien Klin Wochenschr.

[18] Davies A, Jones D, Bailey M, Beca J, Bellomo R, Australia and New Zealand Extracorporeal Membrane Oxygenation Influenza Investigators (2009). Extracorporeal Membrane Oxygenation for 2009 Influenza A(H1N1) Acute Respiratory Distress Syndrome. JAMA.

[19] Group RC, Horby P, Lim WS, Emberson JR, Mafham M, Bell JL (2020). Dexamethasone in hospitalized patients with Covid-19. preliminary report. N Engl J Med..

[20] Gabarre P, Dumas G, Dupont T, Darmon M, Azoulay E, Zafrani L (2020). Acute kidney injury in critically ill patients with COVID-19. Intensive Care Med..

[21] Yang X, Jin Y, Li R, Zhang Z, Sun R, Chen D (2020). Prevalence and impact of acute renal impairment on COVID-19: a systematic review and meta-analysis. Crit Care.

[22] Joannidis M, Forni LG, Klein SJ, Honore PM, Kashani K, Ostermann M (2020). Lung-kidney interactions in critically ill patients: consensus report of the Acute Disease Quality Initiative (ADQI) 21 Workgroup. Intensive Care Med.

[23] Bellelli G, Rebora P, Valsecchi MG, Bonfanti P, Citerio G (2020). members C‑MT. Frailty index predicts poor outcome in COVID-19. patients. Intensive Care Med..

[24] Myers LC, Parodi SM, Escobar GJ, Liu VX (2020). Characteristics of hospitalized adults with COVID-19 in an integrated health care system in california. JAMA.

[25] Huang C, Wang Y, Li X, Ren L, Zhao J, Hu Y (2020). Clinical features of patients infected with 2019 novel coronavirus in Wuhan, China. Lancet.

[26] Arentz M, Yim E, Klaff L, Lokhandwala S, Riedo FX, Chong M (2020). Characteristics and outcomes of 21 critically ill patients with COVID-19 in Washington state. JAMA.

[27] Richardson S, Hirsch JS, Narasimhan M, Crawford JM, McGinn T, Davidson KW (2020). Presenting characteristics, comorbidities, and outcomes among 5700 patients hospitalized with COVID-19 in the New York city area. JAMA.

[28] Vergano M, Bertolini G, Giannini A, Gristina GR, Livigni S, Mistraletti G (2020). Clinical ethics recommendations for the allocation of intensive care treatments in exceptional, resource-limited circumstances: the Italian perspective during the COVID-19 epidemic. Crit Care.

[29] Azoulay E, Beloucif S, Guidet B, Pateron D, Vivien B, Le Dorze M (2020). Admission decisions to intensive care units in the context of the major COVID-19 outbreak: local guidance from the COVID-19 Paris-region area. Crit Care.

