# Supplementary material for: Structured ICU resource management in a pandemic is associated with favorable outcome in critically ill COVID‑19 patients
Source: Wien Klin Wochenschr. 2020 Nov 10;132(21):653–63. doi: 10.1007/s00508-020-01764-0 (PMC7653454; doi:10.1007/s00508-020-01764-0)
Supplement: Supplementary file 1 — A list of COVID-19 ICUs who participated in the Tyrol-CoV-ICU-Reg and ESM Tables 1–3 and ESM Figure 1 are provided in the electronic supplemental material [file 508_2020_1764_MOESM1_ESM.pdf]

Supplementary Appendix

Structured ICU resource management in a pandemic is associated with good outcome in critically ill COVID-19 patients

ESM Tables 1-3
ESM Figure 1

List of COVID-19 intensive care units (ICUs) in Tyrol, Austria who participated in the Tyrolean COVID-19 Intensive Care Registry (Tyrol-CoV-ICU-Reg):

- Medical University Innsbruck, Innsbruck, Austria
    - Medical ICU, Department of Internal Medicine
    - Neurosurgical ICU, Department of Neurosurgery
    - Transplantation ICU, Department of General and Surgical Intensive Care Medicine/Department of Anesthesia and Critical Care Medicine
    - Recovery room "OZA" (adapted as temporary ICU), Department of General and Surgical Intensive Care Medicine/Department of Anesthesia and Critical Care Medicine
    - Recovery room "KHZ" (adapted as temporary ICU), Department of General and Surgical Intensive Care Medicine/Department of Anesthesia and Critical Care Medicine
- Hospital Hall, Hall, Austria
    - ICU, Department of Anesthesia and Intensive Care Medicine
- Hospital Kufstein, Kufstein, Austria
    - ICU, Department of Anesthesia and Intensive Care Medicine
- Hospital Lienz, Lienz, Austria
    - ICU, Department of Anesthesia and Intensive Care Medicine
- Hospital Reutte, Reutte, Austria
    - ICU, Department of Anesthesia and Intensive Care Medicine
- Hospital Schwaz, Schwaz, Austria
    - ICU, Department of Anesthesia and Critical Care Medicine
- Hospital St. Johann i.T., St. Johann in Tyrol, Austria
    - ICU, Department of Anesthesia and Intensive Care Medicine
- Hospital Zams
    - Medical ICU, Department of Internal Medicine
    - Surgical ICU, Department of Anesthesiology and Critical Care Medicine

ESM Table 1 Comorbidities and risk factors

	Overall	ICU survivors	ICU non-survivors	p
n	106	83	23	
**Comorbidities**				
Cardiovascular (%)	45 (42.5)	29 (34.9)	16 (69.6)	0.006
Arterial hypertension (%)	71 (67.0)	54 (65.1)	17 (73.9)	0.583
Renal (%)	21 (19.8)	10 (12.0)	11 (47.8)	<0.001
Liver (%)	7 (6.6)	5 (6.0)	2 (8.7)	1.000
Metastatic disease (%)	0 (0.0)	0 (0.0)	0 (0.0)	NA
Hematological malignancy (%)	3 (2.8)	1 (1.2)	2 (8.7)	0.228
Non hematological malignancy (%)	5 (4.8)	3 (3.6)	2 (9.1)	0.610
Immunosuppression (%)	11 (10.4)	9 (10.8)	2 (8.7)	1.000
COPD (%)	14 (13.2)	10 (12.0)	4 (17.4)	0.748
Asthma (%)	7 (6.6)	5 (6.0)	2 (8.7)	1.000
Respiratory - other (%)	13 (12.3)	8 (9.6)	5 (21.7)	0.228
Neurological (%)	11 (10.4)	9 (10.8)	2 (8.7)	1.000
Diabetes mellitus (%)				0.803
no DM	86 (81.1)	68 (81.9)	18 (78.3)	
Prediabetes	2 (1.9)	1 (1.2)	1 (4.3)	
DM Type I	1 (0.9)	1 (1.2)	0 (0.0)	
DM Type II	16 (15.1)	12 (14.5)	4 (17.4)	
DM (other Type, e.g. MODY)	1 (0.9)	1 (1.2)	0 (0.0)	
BMI [kg/m^2] (%)				0.266
<25	24 (24.2)	21 (27.6)	3 (13.0)	
25<30	51 (51.5)	40 (52.6)	11 (47.8)	
30<35	15 (15.2)	9 (11.8)	6 (26.1)	
35<40	8 (8.1)	5 (6.6)	3 (13.0)	
>40	1 (1.0)	1 (1.3)	0 (0.0)	
Metabolic syndrome (%)	16 (15.1)	10 (12.0)	6 (26.1)	0.182
**Risk factors**				
Smoking within 30 days (%)	11 (11.8)	7 (9.6)	4 (20.0)	0.375
Prior smoking (%)	25 (29.4)	18 (26.9)	7 (38.9)	0.482
ACE-inhibitor (%)	19 (18.3)	16 (19.5)	3 (13.6)	0.747
ARB (%)	19 (18.3)	9 (11.0)	10 (45.5)	0.001
NSAID (%)	14 (14.4)	11 (14.7)	3 (13.6)	1.000
Corticosteroids (%)	13 (12.4)	11 (13.3)	2 (9.1)	0.871
Non-steroidal immunosupressives (%)	7 (6.7)	5 (6.0)	2 (9.1)	0.974

ICU – intensive care unit, COPD – chronic obstructive pulmonary disease, DM – diabetes mellitus, MODY – maturity onset diabetes of the young, BMI – body mass index, ACE – angiotensin-converting enzyme, ARB – angiotensin II receptor blocker, NSAID – nonsteroidal anti-inflammatory drug

ESM Table 2 Lab values available for 49 patients treated at the University Hospital Innsbruck

	Overall	ICU survivors	ICU non-survivors	p
n	49	38	11	
Lowest leukocyte count [G/l] (median [IQR])	6.00 [4.30, 7.40]	5.90 [4.30, 7.07]	7.40 [4.00, 9.75]	0.395
Highest leukocyte count [G/l] (median [IQR])	13.50 [10.90, 17.50]	13.35 [10.12, 16.85]	16.80 [12.95, 19.45]	0.087
Lowest lymphocyte count [%] (median [IQR])	6.50 [4.10, 9.65]	6.85 [4.22, 10.10]	4.50 [2.80, 7.35]	0.142
Lowest thrombocyte count [G/l] (median [IQR])	168.00 [120.00, 216.00]	179.50 [130.00, 213.75]	100.00 [80.00, 218.50]	0.242
Highest thrombocyte count [G/l] (median [IQR])	388.00 [262.00, 462.00]	404.50 [294.75, 482.50]	278.00 [191.00, 320.50]	0.001
Lowest PT [%] (median [IQR])	71.00 [44.75, 88.00]	73.00 [46.00, 91.00]	57.00 [42.00, 77.00]	0.229
Highest PT [%] (median [IQR])	101.50 [96.75, 109.00]	102.00 [97.00, 109.00]	101.00 [93.00, 107.50]	0.676
Highest D-dimer [µg/l] (median [IQR])	11253.50 [5340.50, 33109.00]	9264.00 [3406.00, 20294.50]	34690.00 [11683.50, 35200.00]	0.005
Highest CRP [mg/dl] (median [IQR])	29.27 [17.76, 34.53]	27.11 [16.93, 33.70]	34.53 [28.23, 42.63]	0.018
Highest IL-6 [ng/l] (median [IQR])	744.70 [371.80, 1425.00]	633.85 [270.93, 1217.25]	989.80 [593.15, 3342.50]	0.061
Highest procalcitonin [µg/l] (median [IQR])	1.46 [0.54, 3.05]	1.28 [0.45, 2.07]	4.42 [1.56, 7.39]	0.025
Highest TropT [ng/l] (median [IQR])	38.55 [22.62, 62.10]	26.90 [17.60, 47.75]	63.80 [51.55, 80.50]	0.002
Highest creatinine [mg/dl] (median [IQR])	1.38 [1.02, 1.76]	1.31 [1.00, 1.73]	1.55 [1.28, 2.92]	0.125
Highest cystatin c [mg/l] (median [IQR])	2.01 [1.45, 2.77]	1.63 [1.35, 2.46]	2.23 [1.83, 2.94]	0.124
Highest GOT [U/l] (median [IQR])	106.00 [69.00, 192.00]	94.00 [63.75, 172.25]	154.00 [125.50, 311.00]	0.055
Highest GPT [U/l] (median [IQR])	122.50 [56.00, 214.50]	122.00 [56.00, 206.00]	126.00 [66.00, 256.50]	0.564
Highest AP [U/l] (median [IQR])	229.00 [102.00, 349.50]	220.00 [102.00, 340.00]	275.00 [148.50, 557.50]	0.162
Lowest albumin [mg/dl] (median [IQR])	1817.00 [1606.00, 2115.00]	1824.00 [1518.25, 2089.00]	1754.00 [1673.00, 2154.50]	0.660
Lowest Horovitz (median [IQR])	85.45 [65.62, 118.19]	93.69 [75.14, 126.83]	62.11 [55.90, 73.68]	0.008
Lowest pO2 [mmHg] (median [IQR])	62.25 [54.82, 67.67]	62.90 [56.60, 67.90]	59.00 [52.00, 67.25]	0.345
Highest FiO2 [%] (median [IQR])	72.50 [58.75, 100.00]	70.00 [52.00, 95.00]	100.00 [80.00, 100.00]	<0.001

ICU – intensive care unit, IQR- interquartile range, PT – prothrombin time, CRP – c-reactive protein, IL-6 – interleukin 6, TropT – troponin T, GOT - glutamic oxaloacetic transaminase, GPT - glutamate-pyruvate transaminase, AP – alkaline phosphatase

ESM Table 2 Patient characteristics grouped by Age below and greater than 64 years

	Overall	Age ≤ 64 years	Age > 64 years	p
n	106	65	41	
Age (median [IQR])	64.00 [54.00, 74.50]	57.00 [52.00, 63.00]	76.00 [72.00, 80.00]	<0.001
Male (%)	76 (71.7)	52 (80.0)	24 (58.5)	0.030
BMI (median [IQR])	26.83 [25.07, 29.95]	26.83 [25.11, 29.93]	26.85 [24.79, 29.78]	0.827
HbA1c [%] (median [IQR])	6.20 [5.70, 6.70]	6.15 [5.70, 6.68]	6.20 [5.90, 6.80]	0.325
SAPS III (median [IQR])	56.00 [49.00, 64.00]	50.00 [46.00, 61.25]	61.00 [56.00, 68.00]	<0.001
Pat. from nursing home (%)	0 (0.0)	0 (0.0)	0 (0.0)	NA
Legal guardian (%)	2 (1.9)	1 (1.5)	1 (2.4)	1.000
Admission state (%)				0.007
Fully independent daily living	91 (85.8)	61 (93.8)	30 (73.2)	
Requires some assistance in daily activities	12 (11.3)	4 (6.2)	8 (19.5)	
Requires full assistance in daily activities	3 (2.8)	0 (0.0)	3 (7.3)	
Hospital LOS [days] (median [IQR])	27.00 [13.25, 49.50]	29.00 [18.00, 54.00]	22.00 [9.00, 48.00]	0.119
ICU LOS [days] (median [IQR])	18.50 [5.25, 31.75]	19.00 [11.00, 32.00]	12.00 [5.00, 25.00]	0.309
Death in ICU (%)	23 (21.7)	6 (9.2)	17 (41.5)	<0.001
Death in hospital (%)	24 (22.6)	6 (9.2)	18 (43.9)	<0.001
AKI (%)				0.100
no AKI	54 (50.9)	39 (60.0)	15 (36.6)	
KDIGO I	16 (15.1)	9 (13.8)	7 (17.1)	
KDIGO II	9 (8.5)	5 (7.7)	4 (9.8)	
KDIGO III	27 (25.5)	12 (18.5)	15 (36.6)	
RRT (%)	21 (19.8)	12 (18.5)	9 (22.0)	0.850
IMV (%)	72 (67.9)	46 (70.8)	26 (63.4)	0.564
vv-ECMO (%)	6 (5.7)	6 (9.2)	0 (0.0)	0.116
IMV [days] (median [IQR])	15.00 [10.75, 24.00]	15.50 [11.00, 24.00]	15.00 [9.25, 22.50]	0.435
NIV [days] (median [IQR])	3.00 [1.00, 6.00]	3.00 [1.00, 6.00]	4.00 [1.75, 6.00]	0.328
NHF [days] (median [IQR])	1.00 [1.00, 1.50]	1.00 [1.00, 2.00]	1.00 [1.00, 1.00]	0.618
Prone positioning [days] (median [IQR])	4.00 [2.00, 5.75]	5.00 [2.00, 6.00]	3.00 [1.00, 5.00]	0.110
Continuous muscle relaxation [days] (median [IQR])	1.00 [1.00, 2.00]	1.00 [1.00, 2.00]	1.00 [1.00, 1.00]	0.296
RRT [days] (median [IQR])	11.00 [3.00, 26.00]	17.50 [9.00, 26.50]	8.00 [3.00, 11.00]	0.154
vv-ECMO [days] (median [IQR])	12.00 [11.25, 14.25]	12.00 [11.25, 14.25]	NA [NA, NA]	NA

IQR – interquartile range, BMI – body mass index, HbA1c – glycated hemoglobin, SAPS – simplified acute physiology score, ICU – intensive care unit, LOS – length of stay, AKI – acute kidney injury, KDIGO – kidney disease: improving global outcomes, RRT - renal replacement therapy, vv-ECMO – veno-venous extracorporeal membrane oxygenation, IMV – invasive mechanical ventilation, NIV – non-invasive ventilation, NHF- nasal high flow

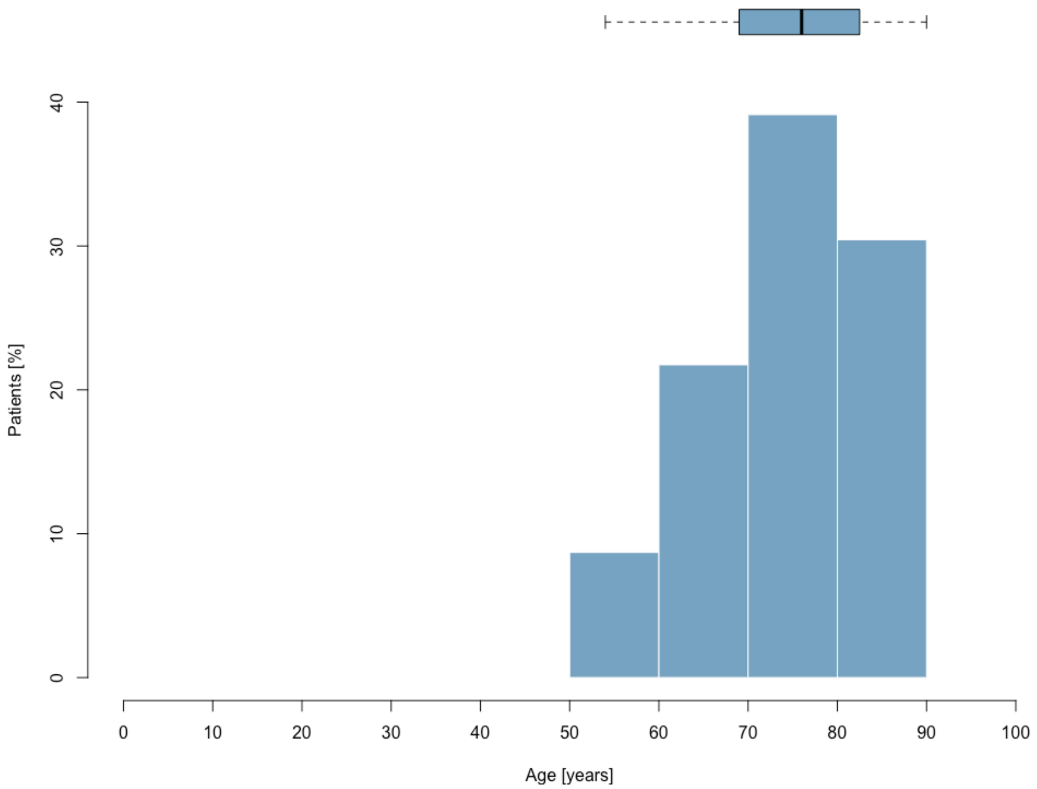

ESM Figure 1 Age ICU non-survivors
